# Interaction Between Chinese Medicine and Warfarin: Clinical and Research Update

**DOI:** 10.3389/fphar.2021.751107

**Published:** 2021-09-20

**Authors:** Wei Zhuang, Shaoli Liu, Xusheng Zhao, Nan Sun, Tao He, Yali Wang, Beibei Jia, Xiaolan Lin, Yanqi Chu, Shengyan Xi

**Affiliations:** ^1^ Department of Pharmacy, Xuanwu Hospital of Capital Medical University, National Gerontic Disease Clinical Research Center, Beijing, China; ^2^ Department of Pharmacy, Haiyang People’s Hospital, Haiyang, China; ^3^ Department of Pharmacy, Beijing Mentougou District Hospital, Beijing, China; ^4^ Department of Pharmacy, Eye Hospital China Academy of Chinese Medical Sciences, Beijing, China; ^5^ Department of Pharmacy, Beijing Hepingli Hospital, Beijing, China; ^6^ Department of Traditional Chinese Medicine, School of Medicine, Xiamen University, Xiamen, China

**Keywords:** Chinese medicine, warfarin, adverse reaction, drug interactions, Chinese patent medicine

## Abstract

**Background:** Warfarin is a commonly used oral anticoagulant. It has a narrow therapeutic window and wide variation in individualized dosing, and is used clinically for the treatment of thromboembolic diseases. Due to the widespread use of traditional Chinese medicine (TCM) in China and the complex composition and diverse mechanisms of action of TCM, the combination of TCM and warfarin in patients has led to fluctuations in the international normalized ratio of warfarin or bleeding. To ensure rational clinical use, we summarize the TCMs with which warfarin interacts and the possible mechanisms, with a view to providing a clinical reference.

**Aim of the study:** To summarize the mechanisms by which Chinese herbal medicines affect the enhancement or weakening of the anticoagulant effect of warfarin, to provide theoretical references for clinicians and pharmacists to use warfarin safely and rationally, and to avoid the adverse effects associated with the combination of Chinese herbal medicines and warfarin.

**Methods:** A computerized literature search of electronic databases, including PubMed, MEDLINE, Cochrane Library, Web of Science (WOS), China National Knowledge Infrastructure (CNKI) and WANFANG Data was performed. Key words used in the literature search were “warfarin”, “Chinese medicine”, “traditional Chinese medicine”, “Chinese patent medicine” etc. and their combinations in a time limit from January 1, 1990 to May 1, 2021. A total of 64 articles were obtained following the selection process, including clinical reports, pharmacological experiments and *in vitro* experiments which were reviewed to determine the mechanism of the anticoagulant effect of herbal medicine on warfarin.

**Results:** The mechanisms affecting the anticoagulant effect of warfarin are complex, and herbal medicines may enhance and diminish the anticoagulant effect of warfarin through a variety of mechanisms; thus, clinical use needs to be cautious. Some herbal medicines have shown inconsistent results in both *in vivo* and *ex vivo* experiments, pharmacology and clinical studies, and should be the focus of future research.

**Conclusion:** With the widespread use of TCM, the combination of warfarin and TCM is more common. This article will promote clinicians’ knowledge and understanding of the TCMs which interact with warfarin, in order to avoid the occurrence of adverse clinical treatment processes, and improve the efficacy and safety.

## Introduction

Warfarin, also known as benzylacetone coumarin sodium, warfarin sodium, etc., is a bicoumarin-like ingredient and was first extracted from wild clover by Wisconsin Alumni Foundation-funded chemist Karl Paul Link in 1940, and was officially approved by the Food and Drug Administration (FDA) in 1954 for use as an oral anticoagulant, and is still used (Wong et al., 2010). Warfarin is currently used for the treatment of primary and secondary prevention of venous thromboembolic disease, prevention of thromboembolism in atrial fibrillation (AF), valvular disease, prosthetic valve replacement, and intracardiac thrombosis. In clinical practice, warfarin is often used in combination with Chinese medicinal herbs and proprietary Chinese medicines for the treatment of thromboembolic diseases. However, due to the complex composition and various mechanisms of action of Chinese medicines, coupled with the narrow therapeutic window of warfarin and large differences in individual doses, the prothrombin time (PT) and international normalized ratio (INR) are often used as its anticoagulation monitoring indices. In recent years, there have been many reports on the interaction between Chinese medicine and warfarin. In this article, we will summarize the mechanism of the enhancement or weakening of the anticoagulant effect of Chinese medicines on warfarin from clinical reports, pharmacological experiments and *in vitro* experiments to provide a theoretical reference for clinicians and pharmacists to use warfarin safely and reasonably and avoid adverse effects of the combination of Chinese medicine and warfarin (See Table 1).

**TABLE 1:** The mechanism of the enhancement or weakening of the anticoagulant effect of Chinese medicines on warfarin.

	Drug name	Sources of drug	Experimental drug	Study subjects	Mechanism of action
**INR↑**	*Salvia miltiorrhiza Bunge*	The dried radix et rhizome of *Salvia miltiorrhiza* Bunge, family *Labiatae*	*Salvia miltiorrhiza* Bunge aqueous extract Li et al. (2016a), Danshen Injection (National Approval No. 991203, produced by Shanghai First Biochemical Pharmaceutical Factory) Xie et al. (2009)	Rat Xie et al. (2009), Li et al. (2016a)	Inhibit platelet aggregation Xie et al. (2009), Li et al. (2016a), increase coagulation factor III and fibrinolytic activity, increase steady-state warfarin blood levels in rats, and decrease warfarin clearance Li et al. (2016a)
			Ethyl acetate extract of *Salvia miltiorrhiza*, aqueous extract of tanshinin; cryptotanshinone, dihydrotanshinone, tanshinone I, tanshinone IIA Purchased from Chengdu Kangjian Biotechnology Co. Qiu et al. (2008), Wang (2015)	*In vitro*/human liver microsomes Qiu et al. (2008), Wang (2015)	Inhibit CYP2C9, CYP1A2 and reduce plasma protein binding of warfarin Qiu et al. (2008), Wang (2015)
	*Carthamus tinctorius* L	The dried flowers of *Carthamus tinctorius* L., family *Asteraceae*	Safflower yellow pigment (National Approval No. 070805, produced by Shenyang Zhonghai Biotechnology Development Co., Ltd. Zhao et al. (2009)	*In vitro*/rabbit	Inhibition of platelet aggregation Liu et al. (2000), Zhao et al. (2009)
			*Carthamus tinctorius* L. extract provided by Shandong Natural Medicines Engineering Technology Research Center Liu et al. (2000)	*In vivo*/rat Liu et al. (2000), Zhao et al. (2009)	
	*Panax notoginseng* (Burkill) F.H.Chen	Dried root of *Panax notoginseng* (Burk.) F.H. Chen, family *Pentacaceae*	*Panax notoginseng* Total Saponin Injection (National Approval No. 081k05, produced by Kunming Pharmaceutical Group Co., Ltd.) Li et al. (2009)	Rat Xu et al. (1997), Li et al. (2009)	Inhibit the activity of CYP2C9 and CYP1A2 Li et al. (2009)
			*Panax ginseng* saponin Rg1 Xu et al. (1997)		Increase intraplatelet cAMP content and decrease thromboxane A-2 Xu et al. (1997)
	Shunaoxin Dripping Pills	Composed of *Conioselinum anthriscoides* ‘Chuanxiong’ and *Angelica sinensis* (Oliv) Diels	National Approval No. 672023, produced by Tianjin Zhongxin Pharmaceutical Group Co., Ltd. 6th Traditional Chinese Medicine Factory Feng et al. (2015)	*In vivo*, *in vitro*/rat blood Feng et al. (2015)	Anti-platelet aggregation Feng et al. (2015)
	*Lycium barbarum L*	The ripe fruit of *Lycium barbarum* L., family *Solanaceae*	*Lycium barbarum* L. juice	*In vitro*/human liver microsomes Leung et al. (2008)	Inhibition of CYP2C9 enzyme activity Leung et al. (2008)
			*Lycium barbarum* polysaccharide provided by Shaanxi CiYuan Biotechnology Co., Ltd. Cheng (2012)	*In vitro*/cellular Cheng (2012)	Competition for P-glycoprotein substrates Cheng (2012)
	*Glycyrrhiza glabra* L	Dried roots and rhizomes of *Glycyrrhiza glabra* L., a genus of licorice in the *Legume* family	*Glycyrrhiza glabra* L.aqueous extract Xiang et al. (2008), Chen et al. (2018)	Rat Xiang et al. (2008)	Inhibit CYP1A2, 2C9, 2C19, 2D6 and 3A4/5 activity and inhibit platelet function in multiple sessions Xiang et al. (2008)
				*In vivo*/human Chen et al. (2018)	Antiplatelet activity of coumarin derivatives Chen et al. (2018)
	*Curcuma longa* L	Dried rhizome of *Curcuma longa* L., family *Zingiberaceae*	*Curcuma longa* L.distillation extract Xia et al. (2012)	*In vivo*/mouse blood Xia et al. (2012)	Inhibition of the CYP3A isoform of the CYP45016 enzyme system Xia et al. (2012)
				*In vitro*/human blood Xia et al. (2012)	
	*Eleutherococcus senticosus* (Rupr. & Maxim.) Maxim	The rhizome or stem of *Eleutherococcus senticosus* (Rupr. & Maxim.) Maxim., family *Cinchona*	*Eleutherococcus senticosus* (Rupr. & Maxim.) Maxim. Injection (National Approval No. 20110207, produced by Heilongjiang Ursuline Pharmaceutical Co., Ltd.) Zeng et al. (2012)	*In vitro*/rat liver microsomes Zeng et al. (2012)	Inhibition of CYP2C9 and CYP3A4 Zeng et al. (2012)
	*Conioselinum anthriscoides* ‘Chuanxiong'	Dried rhizome of *Conioselinum anthriscoides* ‘Chuanxiong’ in the family *Umbelliferae*	*Conioselinum anthriscoides* ‘Chuanxiong’ aqueous extract Li et al. (2016b)	Rat Li et al. (2016b)	Inhibition of CYP2C9 and CYPlA2 Li et al. (2016b)
	*Silybum marianum* (L.) Gaertn	The dried ripe fruit of *Silybum marianum* (L.) Gaertn., family *Asteraceae*	*Silybum marianum* (L.) Gaertn. Extract Brantley et al. (2010)	*In vivo*/human liver microsomes Brantley et al. (2010)	Moderate inhibition of CYP1A2, CYP2C8, CYP2C9 enzymes and weak inhibition of CYP2A6, CYP2C19, CYP2D6 and other enzymes Brantley et al. (2010)
	*Ginkgo biloba* extract	The main medicinal parts of *Ginkgo biloba* L, leaves and fruit	*Ginkgo biloba* extract tablet (produced by Shonan Guohua Pharmaceutical Co., Ltd.) Tan et al. (2011)	*In vitro*/human Tan et al. (2011)	Inhibit platelet activation and aggregation Tan et al. (2011)
			*Ginkgo biloba* extract (produced by Guizhou Yibai Pharmaceutical Co., Ltd.) Zhou and Zeng (2011)	Rat Zhou and Zeng (2011)	Affect the enzyme activity of CYP2C9 Zhou and Zeng (2011)
	*Coptis chinensis* Franch. and *Phellodendron amurense* Rupr.	The rhizome and root of the berberine plant *Plagiorhegma dubia* Maxim.; the dried bark of *Phellodendron chinense*, a genus of *Phellodendron* in the family *Rutaceae*	Berberine hydrochloride (produced by National Institute for the Control of Pharmaceutical and Biological Products) Tan et al. (2002)	*In vitro/in vitro* dialysis of berberine hydrochloride to replace warfarin Tan et al. (2002)	Compete to bind Warfarin Binding Plasma Protein Tan et al. (2002)
				Mouse Tan et al. (2002)	
	Fufang Danshen Dripping Pills	*Salvia miltiorrhiza* Bunge, *Panax notoginseng*, and borneol. et al	National Approval No.Z10950111produced by Tasly Pharmaceutical Group Co., Ltd. Chu et al. (2011), Yi et al. (2013)	*In vitro*/human Yi et al. (2013)	Decrease in absorption rate, apparent volume of distribution, and elimination half-life Chu et al. (2011), Yi et al. (2013)
				Rat Chu et al. (2011)	
	Danhong Injection	*Salvia miltiorrhiza* Bunge and *Carthamus tinctorius* L.	National Approval No.Z20026866 produced by Shandong Danhong Pharmaceutical Co., Ltd. Feng et al. (2016)	Rat Feng et al. (2016)	Exogenous coagulation pathway enhances the anticoagulation of warfarin Feng et al. (2016)
	*Pyrola calliantha* Andres calliantha Andres	The whole plant of *Pyrola rotundifolia* L. subsp. *chinensis* H. Andres, *P. decorata* H. Andres or *P. rotundifolia* L	Salicylic acid constituents of *Pyrola* Xie et al. (2001)	*In vitro*/human Xie et al. (2001)	Inhibit the oxidoreductase of vitamin K and hinder its metabolic cycle; salicylic acids compete for the binding of warfarin plasma protein Xie et al. (2001)
	*Tribulus terrestris* L	The mature fruit of *Tribulus terrestris* L., a plant of the family *Tribulus terrestris*	*Tribulus terrestris* L. preparation drugs Tang (2007)	*In vitro*/human Tang (2007)	The mechanism of action is unclear, and further research is needed Tang (2007)
	*Angelica sinensis* (Oliv) Diels	The root of *Angelica sinensis* (Oliv) Diels. in the family *Umbelliferae*	*Angelica sinensis* (Oliv) Diels. water extract Lo et al. (1995), Ye et al. (2014)	Rabbit Lo et al. (1995)	Inhibit the formation of thromboxane and platelet aggregation, and compete with warfarin plasma protein binding Lo et al. (1995)
				*In vitro*/human Ye et al. (2014)	Contain coumarin-based active ingredients Ye et al. (2014)
**INR↓**	*Hypericum perforatum*	The dried above-ground part of *Hypericum perforatum* L., family *Garciniaceae*	*St John’s* Wort preparation (300 mg tablet, 0.3% hypericin, produced by HWI Analytik, Gmhb, Ruelzheim, Germany) Wang et al. (2009)	*In vitro*/human Wang et al. (2009), Silva et al. (2016)	Induce CYP3A4 activity Wang et al. (2009)
			*Hypericum perforatum* extract Silva et al. (2016)	*In vitro*/human hepatocytes Silva et al. (2016)	Induce CYP1A2 activity and promote warfarin metabolism Silva et al. (2016)
	*Panax ginseng* C.A.Mey. and *Panax quinquefolius* L	The dried roots of *Panax ginseng* C. A. Mey. of the family *Pentacostaceae*; the root of *Panax quinquefolius* L., a plant in the *Araliaceae* family	Ginseng 500 mg capsules (Contains ginseng whole root powder, from Vitamin Lab) Malati et al. (2012)	*In vitro*/human Malati et al. (2012)	Ginsenoside F1 induces CYP3A gene expression and enhances its enzyme activity Malati et al. (2012)
			Ginsenoside Hao et al. (2008)	Fluorescence Probe Method Hao et al. (2008)	Ginsenosides Rg3, Rh2, C-K can inhibit the activity of CYP2C9, CYP3A4 and CYP2C19, Rb1, C-K can moderately inhibit the activity of CYP1A2, and the mechanism of weakening the anticoagulant effect of warfarin remains to be further clarified Hao et al. (2008)
	*Ginkgo biloba* L	Leaves of *Ginkgo biloba*, a genus of the *Ginkgo* family	*Ginkgo biloba* preparations Zhang et al. (2007)	Clinical Zhang et al. (2007)	Induce hepatic cytochrome P450 enzymes, accelerate the metabolism of warfarin Zhang et al. (2007)

## Methods

A computerized literature search of the electronic databases, including PubMed, MEDLINE, Cochrane Library, Web of Science (WOS), China National Knowledge Infrastructure (CNKI) and WANFANG Data was performed. Key words used in the literature search were “warfarin”, “Chinese medicine”, “traditional Chinese medicine”, “Chinese patent medicine” etc. and their combinations in a time limit from January 1, 1990 to May 1, 2021. A total of 64 articles were obtained following the selection process, including clinical reports, pharmacological experiments and *in vitro* experiments which were reviewed to determine the mechanism of the anticoagulant effect of herbal medicine on warfarin.

## Results

### Warfarin Anticoagulation Mechanism and Metabolism

#### Warfarin Anticoagulation Mechanism

Warfarin is a vitamin K carboxylase, so that the synthesis of coagulation factors II, VII, IX and X during the process of glutamate carboxylation is inhibited, these vitamin K-dependent coagulation factors can only be activated in the precursor stage, in order to achieve the anticoagulant effect. However, warfarin has no inhibitory effect on coagulation factors already synthesized by the liver and has to wait for the concentration of coagulation factors to decrease before it can act. In addition, vitamin K antagonists inhibit the carboxylation of anticoagulant protein C and protein S.

#### Metabolism of Warfarin

Warfarin sodium is >90% bioavailable and reaches peak plasma concentration at 3–9 h. Warfarin sodium is heavily bound to albumin, with the free fraction ranging from 0.5 to 3%. The volume of distribution is approximately 0.14 L/kg. Warfarin sodium may enter the placenta but is not excreted into the emulsion. Warfarin sodium is cleared by hepatic metabolism to inactive metabolites excreted in the urine by the hepatic microsomal enzymes CYP2C9 (S-warfarin) and by CYP1A2 and CYP3A (R-warfarin). The clearance half-life of S-warfarin sodium is 18–35 h and that of R-warfarin sodium is 20–70 h.

### Analysis of the Mechanisms Affecting the Anticoagulant Effect of Warfarin

There are many factors affecting the anticoagulant effect of warfarin, which have been analyzed in a large number of studies, and the following five aspects have been determined from the perspective of mechanism: *a.* Influences platelet aggregation response. *b.* Influences warfarin metabolism. *c.* Influences warfarin plasma protein binding rate. *d.* Affects the action of the warfarin antagonist vitamin K. *e.* Contains coumarin-like components similar to warfarin (Huang and Jin, 2011).

#### Varieties and Mechanisms of Traditional Chinese Medicines to Enhance the Anticoagulant Effect of Warfarin

##### Reduce Platelet Aggregation

*Salvia miltiorrhiza* Bunge (Danshen): The dried radix et rhizome of *Salvia miltiorrhiza* Bunge has the functions of promoting blood circulation and removing blood stasis, relieving pain by dredging meridians, clearing heart fire, relieving restlessness, and cooling blood to diminish carbuncle. Modern pharmacological effects are anti-coagulation, anti-thrombosis, improvement of microcirculation, improvement of blood rheology, anti-myocardial ischemia, anti-cerebral ischemia, anti-oxidation, etc. *In vivo* (Fan et al., 2010), salvianolic acid A (SAA) did not affect coagulation parameters in rats after intravenous administration for five successive days. *In vitro*, pretreatment with SAA on washed rat and human platelets significantly inhibited various agonists, stimulated platelet aggregation and caused an increase in cyclic adenosine monophosphate (cAMP) level in platelets activated by adenosine diphosphate (ADP). SAA possesses antithrombotic activities. The antithrombotic effect might be related to its antiplatelet action and ability to modulate hemorheology without affecting the coagulation system. The mechanisms underlying such activities may involve the induction of cAMP. Li Le reported that Salvia miltiorrhiza can increase the activity of coagulation factor Ⅲ and fibrinolysis by inhibiting platelet aggregation, and can reduce the clearance rate of warfarin to enhance its anticoagulant effect (Li L. et al., 2016). A pharmacological study by Xie H.J. et al. showed that Salvia miltiorrhiza has the effect of anti-platelet aggregation, but does not affect blood coagulation function in rats (Xie et al., 2009). When combined with warfarin, it can increase the steady-state blood concentration and INR of warfarin in rats, and enhance the anticoagulation of warfarin.

*Carthamus tinctorius* L. (Honghua): The dried flower of *Carthamus tinctorius* L has the functions of promoting blood circulation and menstruation, relieving pain by removing blood stasis, and modern pharmacological studies have shown that it has anticoagulation and antiplatelet aggregation effects (Liu et al., 2012; Xu and Dong, 2012). Studies by Zhao et al. (2009) have shown that *Carthamus tinctorius* L. extract had a significant inhibitory effect on ADP-induced platelet aggregation in rabbits. It can significantly inhibit platelet aggregation *in vivo* and *in vitro*, and has a certain inhibitory effect on the activation of the endogenous coagulation system. It may produce a synergistic anticoagulant effect with warfarin. Studies by Liu et al. (2000), Zang et al. (2007), and Zhang et al. (2013) showed that safflower yellow, the main active ingredient in *Carthamus tinctorius* L., can inhibit platelet adhesion, 5-hydroxytryptamine (5-HT) release and increase the concentration of free Ca^2+^ induced by platelet activating factor, significantly improve blood coagulation function, and has anti-thrombotic effects. Zhang et al. (2016) studies in rats showed that the combined use of *Carthamus tinctorius* L. and warfarin significantly prolonged PT value and bleeding time, but has no effect on the blood concentration of warfarin.

*Panax notoginseng* (Burkill) F.H. Chen (Sanqi): The dry radix et rhizome of *Panax notoginseng* (Burk.) F.H. Chen has the effects of removing blood stasis, stopping bleeding, promoting blood circulation and relieving pain. Modern pharmacological effects include hemostasis (promoting blood clotting), anti-thrombosis, promoting hematopoiesis, inhibiting the heart, expanding blood vessels, and lowering blood pressure. The main components of *Panax notoginseng* are total saponins (*Panax notoginseng* saponins, PNS). PNS mainly consists of *Panax notoginseng* saponin Rg1 and *Panax notoginseng* saponin Rb1. It is widely used in the clinical treatment of various cerebrovascular diseases. When combined with warfarin, it can increase the peak concentration of warfarin to enhance its anticoagulant effect, and significantly increase the PT value and INR value of warfarin (*p* < 0.05). The mechanism underlying the reduction of thrombosis may be through increasing the cAMP content in platelets and reducing thromboxane A-2 (TXA-2) (Xu et al., 1997). Therefore, *Panax notoginseng* combined with warfarin may cause increased INR and multiple subcutaneous hemorrhage and ecchymosis.

Shunaoxin Dripping Pills: Shunaoxin Dripping Pills are composed of Ligusticum chuanxiong and Angelica sinensis (42 mg/pill, Tianjin Zhongxin Pharmaceutical Group Co., Ltd. Sixth Chinese Medicine Factory). They have the effects of regulating qi, promoting blood circulation, removing blood stasis and relieving pain. Studies by Feng Bo (Feng et al., 2015) have shown that Shunaoxin Dripping Pills have a strong anti-platelet aggregation effect *in vitro*, and with an increase in dose, the inhibition of platelet aggregation *in vivo* increased slightly. Shunaoxin Dripping Pills can significantly reduce platelet aggregation induced by ADP and thrombin, and is positively correlated with the dose. High doses combined with warfarin can significantly prolong APTT, PT, and thrombin time (TT), suggesting that Shunaoxin Dripping pills have the effect of anti-platelet aggregation, and high-dose combination with warfarin can increase the anticoagulant effect of warfarin.

##### Reduction of Warfarin Metabolism

*Salvia miltiorrhiza* Bunge (Danshen): Wang et al. (2010a) studied human cells and showed that tanshinone I, tanshinone IIA and cryptotanshinone strongly inhibited CYP1A2, moderately inhibited CYP2C9, tanshinone I and cryptotanshinone, weakly inhibited CYP3A4, dihydrotanshinone and competitively inhibited CYP1A2 and CYP2C9, but had no effect on CYP3A4. Qiu et al. (2008), Wang (2015) investigated the effects of various components of *Salvia miltiorrhiza* Bunge on the activity of cytochrome P450s (CYPs) in humans and found that cryptotanshinone, tanshinone I and tanshinone IIA competitively inhibited CYP1A2, cryptotanshinone and tanshinin moderately inhibited CYP2C9, protocatechualdehyde slightly inhibited CYP3A4, while the lipid soluble components of *Salvia miltiorrhiza* Bunge could induce CYP3A4. Wang et al. (2009), Wang et al. (2010b) showed in both *in vivo* and *ex vivo* experiments in rats that tanshinones inhibited the activity of CYP2C11, but had fewer pharmacodynamic effects on the CYP2C11-specific substrate toluene sulfonylurea, which had the same specific metabolic substrate as human CYP2C9, therefore it can be concluded that various components of *Salvia miltiorrhiza* Bunge may affect the anticoagulant effect of warfarin by inhibiting CYP2C9 and CYP1A2, inhibiting or inducing CYP3A4, and competitively binding to human albumin and other ways. Wu and Yeung (2010) also found that tanshinone reduced CYP1A1, CYP2C6 and CYP2C11 mediated 4-, 6- and 7-hydroxywarfarin hydroxylation reactions in rats, thereby inhibiting warfarin metabolism. Zhou et al. (2012) also concluded that *Salvia miltiorrhiza* Bunge affected the anticoagulant effect of warfarin in relation to CYP450 enzyme metabolism.

*Lycium barbarum* L. (Gouqizi): The mature fruit of *Lycium barbarum* L., family *Solanaceae* has the effect of nourishing the liver and kidney, benefiting the essence and brightening the eyes, treating liver and kidney yin deficiency and premature aging. It has anti-aging, hypolipidemic, hypoglycemic and hematopoietic effects. In an *in vitro* study (Leung et al., 2008), noted that *Lycium barbarum* L. tea inhibited warfarin metabolism by weakly inhibiting CYP2C9 activity in human liver microsomes, suggesting that this interaction may be due to the effect of factors such as absorption, P-glycoprotein or the anticoagulant effect of the herb itself. It was also observed that it was possible that the metabolites of *Lycium barbarum* L. displaced warfarin from its plasma protein binding site, leading to an increase in INR. Cheng (2012) confirmed using a Caco-2 cell assay that *Lycium barbarum* polysaccharides (LBP) may increase the absorption of the drug when combined with P-glycoprotein substrate, resulting in a higher blood concentration and enhanced efficacy. The high concentration of LBP increased the absorption of the drug by inhibiting the efflux transport of P-glycoprotein and increased the blood concentration and enhanced the effect of warfarin. Studies (Rivera et al., 2012; Zhuang et al., 2020) reported an unexplained and significant increase in INR value along with symptoms of rhinorrhagia, skin petechiae and rectal bleeding in an American woman after taking *Lycium barbarum* L. juice. Guzmán et al. (2021) reported a 75-year-old female patient who had a mitral valve replacement 3 years ago and was taking oral warfarin postoperatively with an INR controlled within the target range (2.5–3.5). Recent consumption of *Lycium barbarum* L. tea (1-2 cups per day) to prevent neocoronavirus infection eventually led to an increase in INR due to warfarin overdose.

*Glycyrrhiza glabra* L. (Gancao): *Glycyrrhiza glabra* L. is the dried root and rhizome of *Glycyrrhiza uralensis* Fisch., *Glycyrrhiza inflata* Bat. or *Glycyrrhiza glabra* L., a genus of licorice in the family *Fabaceae*. It has the effects of benefiting the qi and tonifying the middle, moistening the lung and relieving cough, clearing heat and detoxifying, relieving pain and harmonizing medicinal properties. It has modern pharmacological effects such as adrenocorticotropic hormone-like effects, anti-platelet aggregation, anti-peptic ulcer, antipyretic, sedative, and immune enhancing. Qiao et al. (Qiao et al., 2014) analyzed the interaction of more than 40 chemical components in *Glycyrrhiza glabra* L. with cytochrome P450 enzymes and showed that the flavonoids, terpenoids and coumarins in *Glycyrrhiza glabra* L. had different degrees of inhibitory effects on the activities of CYP1A2, 2C9, 2C19, 2D6 and 3A4/5, which resulted in slower warfarin metabolism, longer half-life and higher INR. Flavonoids can inhibit platelet function through multiple links (Xiang et al., 2008), mainly including blocking platelet activating factor (PAF) binding to platelet receptors, inhibiting the release of platelet endogenous substances, inhibiting the increase in intra-platelet Ca^2+^ and balancing the intra-platelet thromboxane B2-6-keto-prostaglandin (TXB2-6-keto-PGFla) system, which synergistically anticoagulated with warfarin.

*Curcuma longa* L. (Jianghuang): Jianghuang is the dried rhizome of *Curcuma longa* L., family *Zingiberaceae*. It has the effect of blood circulation activity and pain relief. It has modern pharmacological effects such as antihypertensive, antibacterial, antiviral, hepatoprotective, antioxidant, hypolipidemic and antitumor. *Curcuma longa* L. promotes the synthesis of prostaglandin PGI2, reduces the production of TXA2, and interferes with the production of cAMP or Ca^2+^ in platelets. Xia Q et al. found that *Curcuma longa* L. had a strong inhibitory effect on the CYP3A isoform of the CYP450 enzyme system (Xia et al., 2012). Therefore, *Curcuma longa* L. in combination with warfarin may enhance the anticoagulant effect of warfarin and lead to bleeding; thus, INR values should be monitored during clinical treatment.

*Eleutherococcus senticosus* (Rupr. & Maxim.) Maxim. (Ciwujia): *Eleutherococcus senticosus* (Rupr. & Maxim.) Maxim. is the rhizome or stem of *Acantha panax senticosus* (Rupr. et maxim) Harms, a plant of the genus Wujia, family Wujia. It has the effect of benefiting the qi, strengthening the spleen, tonifying the kidney and calming the mind. It has modern pharmacological effects of excitement or inhibition of the central nervous system, antitussive, expectorant and anti-platelet aggregation. Li et al. (2015) showed that *Eleutherococcus senticosus* (Rupr. & Maxim.) Maxim. injection had no significant effect on APTT, PT and INR in rats, and the Cmax, AUC_0-∞_, t_1/2_, APTT and PT increased when combined with warfarin, suggesting that *Eleutherococcus senticosus* (Rupr. & Maxim.) Maxim. injection itself had no anticoagulant effect, but can affect its pharmacokinetics and anticoagulant effect when combined with warfarin, and weaken warfarin metabolism by inhibiting the effect of CYP2C9 and CYP3A4 (Zeng et al., 2012).

*Conioselinum anthriscoides* ‘Chuanxiong’ (Chuanxiong): This is the dried rhizome of *Ligusticum chuanxiong* Hort of the *Umbelliferae* family. It has the effect of activating blood circulation and qi circulation, dispelling wind and relieving pain. Its active ingredient of ligustrazine can dilate coronary arteries, increase coronary blood flow, improve myocardial blood oxygen supply, and reduce myocardial oxygen consumption; it can reduce platelet surface activity, inhibit platelet agglutination, and prevent thrombus formation. Li et al. (2016b) found that *Conioselinum anthriscoides* ‘Chuanxiong’ extract had inhibitory effects on CYP2C9 and CYPlA2, competitively inhibited metabolic enzymes, increased free warfarin, and increased the anticoagulant effect.

*Silybum marianum* (L.) Gaertn. (Shuifeiji): This is the dried and mature fruit of the *Silybum*, family *Asteraceae*. It is harvested in autumn when the fruit is ripe, impurities are removed and it is dried in the Sun. It is bitter and cool to taste, with the effect of clearing heat and detoxifying, draining the liver and stimulating the bile. The modern pharmacological effects of *Silybum marianum* (L.) Gaertn. include protecting against liver injury, and anti-liver virus. Brantley et al. (2014) found that its combination with warfarin could increase the area under drug time curve (AUC) of S-type warfarin, which enhanced its anticoagulant effect. *Silybum marianum* (L.) Gaertn. can moderately inhibit the activities of CYP1A2, CYP2C8, and CYP2C9 enzymes and weakly inhibit the activities of CYP2A6, CYP2C19, CYP2D6 and other enzymes (Brantley et al., 2010). *Silybum marianum* (L.) Gaertn. can enhance the anticoagulant effect of warfarin by weakening its metabolism when combined with warfarin.

*Ginkgo biloba* extract (GBE): Ginkgo is a genus of *Ginkgo biloba* L. in the family *Ginkgoaceae*, also known as white fruit tree, gongsun tree, and duck palm tree, whose medicinal parts are mainly the leaves and fruits. Ginkgo has the functions of invigorating blood circulation, and removing blood stasis. Modern pharmacological studies have shown that ginkgo has hepatoprotective, antitumor, antiradiation, and renal protective effects, as well as antioxidant, pro-intellectual, anti-anxiety, sedative, lipid regulating and reducing ischemia-reperfusion injury effects (Han et al., 2007; Shi, 2008). Tan et al. (2011) found that *Ginkgo biloba* extract (GBE) could block the production of platelet-derived growth factors through the inhibition of tyrosine kinase, thereby inhibiting thrombin-induced platelet activation and aggregation, with no or minimal effect on coagulation. *In vitro* studies have shown that GBE affects the enzymatic activity of CYP2C9 *in vivo*, thereby affecting S-warfarin 7-hydroxylation. However, *in vivo* studies have shown that GBE has no effect on the metabolism of the CYP2C9 substrates diclofenac and toluenesulfonylurea, and that neither GBE nor ginkgolide B affects the coagulation process, but that ginkgolide attenuates the anticoagulant effect of warfarin by inducing S-warfarin hydroxylase. Some experiments in rats have shown that GBE induces CYPs, but enzyme activity is quickly restored after discontinuation of dosing. GBE had no significant effect on single-dose warfarin pharmacodynamics, no effect on PT and APTT, and no significant change in apparent volume of distribution, but increased peak warfarin concentration, AUC, half-life, and decreased its clearance (Zhou and Zeng, 2011).

*Salvia miltiorrhiza* Bunge (Danshen): Lo et al. (1992) studied the effect of *Salvia miltiorrhiza* Bunge on warfarin metabolism in rats. It was found that *Salvia miltiorrhiza* Bunge decreased the absorption rate, apparent volume of distribution and clearance half-life of warfarin, and increased the peak concentration and time to peak thereby enhancing the anticoagulant effect of warfarin.

Fufang Danshen Dripping Pills: Fufang Danshen Dripping Pills have the effect of activating blood circulation, resolving blood stasis, regulating qi and relieving pain. Fufang Danshen Dripping Pills consist of *Salvia miltiorrhiza* Bunge, *Panax notoginseng*, and borneol. Yi et al. (2013) found that Fufang Danshen Dripping Pills (*Salvia miltiorrhiza* Bunge, *Panax notoginseng*, and borneol, 27 mg/pill, Tianshili Pharmaceutical Group Co., Ltd.) could alter the pharmacokinetic parameters of warfarin. Total *Panax notoginseng* saponin can reduce the metabolic rate of warfarin and enhance its anticoagulant effect by inhibiting its hydroxylation in rats. Yi et al. (2013) found that Fufang Danshen Dripping Pills alone for 4 weeks had a significant effect on coagulation. A significant increase in PT values was observed following the combination of Fufang Danshen Dripping Pills and warfarin, thus it was hypothesized that Fufang Danshen Dripping Pills and warfarin have a synergistic effect on anticoagulation. A clinical study (Guo and Zheng, 2009) found that the Cmax, AUC_0∼144_, AUC_0∼∞_, t_1/2_, PT, and APTT of warfarin were increased in healthy volunteers who were co-administered Fufang Danshen Dripping Pills, suggesting that Fufang Danshen Dripping Pills can affect the metabolism of warfarin and enhance its anticoagulant effect. However, animal experiments have also shown (Chu et al., 2011) that there were no significant changes in warfarin pharmacodynamics and pharmacokinetics when Fufang Danshen Dripping Pills were administered with warfarin, suggesting that regular doses of Fufang Danshen Dripping Pills may have no effect on warfarin pharmacodynamics and pharmacokinetics in humans. Due to inconsistent results between animal experiments and clinical applications, further research is needed.

Danhong Injection: Danhong injection (*Salvia miltiorrhiza* Bunge and *Carthamus tinctorius* L., 10 ml/stem, Shandong Danhong Pharmaceutical Co., Ltd.) significantly increased PT and INR values in rats (*p* < 0.01), and the effect of Danhong injection on APTT values was relatively small (*p* < 0.05). This suggests that Danhong injection has anticoagulant effects. It was shown that the combination of Danhong injection and warfarin could produce pharmacodynamic synergy and enhance the anticoagulant effect of warfarin mainly through the exogenous coagulation pathway, but also had some effect on the endogenous coagulation pathway (Feng et al., 2016). The combination of Danhong injection and warfarin increases the anticoagulant effect of warfarin and also increases the risk of bleeding, and the combination should be avoided or the dose should be reasonably adjusted and monitored.

#### Competitive Binding to Plasma Proteins Increases Free State Warfarin Concentration

*Salvia miltiorrhiza* Bunge (Danshen): Wang Q. et al. (Tan et al., 2002; Liu et al., 2008; Guo and Zheng, 2009; Wang, 2013) concluded that the binding rate of *Salvia miltiorrhiza* Bunge and tanshinone IIa to human serum proteins was comparable to that of warfarin, and could replace warfarin in warfarin-albumin complexes, which could significantly reduce the plasma protein binding rate of warfarin, increase the free warfarin concentration in blood, and enhance the anticoagulant effect. Wang (2013) also found that tanshinone IIa and oleanolic acid in *Salvia miltiorrhiza* Bunge also have certain inhibitory effects on coagulation factor Xa. When *Salvia miltiorrhiza* Bunge is used in combination with warfarin, the anticoagulant effect is enhanced, which may lead to bleeding adverse reactions.

*Coptis chinensis* Franch. (Huanglian) and *Phellodendron amurense* Rupr. (Huangbai): *Coptis chinensis* Franch. is the rhizome and root of the berberine plant *Plagiorhegma dubia* Maxim. *Phellodendron amurense* Rupr. is the dried bark of *Phellodendron chinense*, a genus of *Phellodendron* in the family *Rutaceae*. The active ingredient in both *Coptis chinensis* Franch. and *Phellodendron amurense* Rupr. is berberine, and these have the function of clearing heat and drying dampness, purging fire and detoxifying toxins. Modern pharmacological studies have found that *Coptis chinensis* Franch and *Phellodendron amurense* Rupr. have anti-inflammatory and antibacterial effects. Study (Tan et al., 2002) showed in an *in vitro* balance dialysis assay that the berberine component contained in *Coptis chinensis* Franch. and *Phellodendron amurense* Rupr. can compete to bind plasma proteins and increase the concentration of free warfarin in plasma, enhancing its anticoagulant effect; pharmacodynamics (Tan et al., 2002) indicated that the coagulation time in mice was significantly prolonged when berberine was combined with warfarin (*p* < 0.01), indicating its ability to enhance the anticoagulant effect. A case of subcutaneous petechiae in a patient taking warfarin combined with Huang Lian Qing Huo Wan has also been reported (Geng, 2004).

#### Inhibiting the Effect of Warfarin Antagonist Vitamin K

Vitamin K can antagonize the anticoagulant effect of warfarin, so TCMs containing ingredients that inhibit vitamin K compete with vitamin K in the body, so that the vitamin K-dependent coagulation factors II, VII, IX and X cannot be synthesized in time, thus having an anticoagulant effect and potentiating the anticoagulant effect of warfarin (Hirsh et al., 1998).

*Pyrola calliantha* Andres calliantha Andres (Luticao): This is the whole plant of *Pyrola rotundifolia* L. Subsp. *Chinensis* H. Andres, *P. decorata* H. Andres or *P. rotundifolia* L., also known as deer’s-foot herb. *Pyrola calliantha* Andres has antibacterial, cardiac contractility enhancing, antiarrhythmic and contraceptive effects, and is mainly used in lung infections, intestinal and urinary tract infections, liver abscesses and other diseases (Wang, 1983). Xie et al. (2001) reported that *Pyrola calliantha* Andres, whose active ingredients are salicylates, can affect thrombin synthesis by inhibiting the oxidoreductase enzyme of vitamin K impeding its metabolic cycle and reducing the absorption of vitamin K. It may also be due to salicylates displacing warfarin from plasma protein binding and elevating the blood concentration of warfarin resulting in an enhanced anticoagulant effect of warfarin.

*Tribulus terrestris* L. (Jili): The mature fruit of *Tribulus terrestris* L., a plant of the family *Tribulus terrestris* has the effects of calming the liver and relieving depression, activating the blood and dispelling wind, brightening the eyes, and relieving itching. Modern pharmacological effects include anti-myocardial ischemia, delaying aging, and anti-acetylcholine. Tang (2007) reported that *Tribulus terrestris* L. and its preparations can inhibit the production of vitamin K by intestinal flora, interfere with platelet aggregation, and affect the absorption, distribution, and metabolism of warfarin, so that the anticoagulant effect of warfarin is enhanced, pending further research.

#### Plants Containing Coumarin-like Ingredients Similar to Warfarin, Producing a Synergistic Effect

*Angelica sinensis* (Oliv) Diels. (Danggui): The root of *Angelica sinensis* (Oliv) Diels. in the family *Umbelliferae* has pharmacological effects which include increasing coronary flow, reducing myocardial oxygen consumption, anti-arrhythmia, anti-atherosclerosis, inhibiting platelet aggregation, improving microcirculation, and improving immunity in the body. In 1995 Lo et al. (1995) showed using chemical analysis that Angelica sinensis contains coumarin-like active ingredients similar to warfarin such as parsley oil methyl ether and psoralen. These ingredients can inhibit the formation of thromboxane and platelet aggregation (Liu et al., 1995), and have a synergistic effect when combined with warfarin; in animal experiments, it was concluded that angelica had no effect on PT and pharmacokinetic parameters in warfarin single-dose experiments (*p* > 0.05). However, in warfarin steady-state experiments, angelica prolonged PT in rabbits, but had no effect on warfarin blood concentrations. Another reason may be that warfarin is able to displace angelica from its binding to plasma proteins, thereby increasing the blood concentration of angelica, which in excess produces anticoagulant effects due to the presence of coumarin-like components. Ye Z.W. reported (Ye et al., 2014) that ferulic acid in angelica administered *in vivo* or *in vitro* inhibits PAF, inhibits platelet aggregation reaction and enhances the anticoagulant effect of warfarin.

*Glycyrrhiza glabra* L. (Gancao): The coumarin derivatives in *Glycyrrhiza glabra* L. (Chen et al., 2018) may show antiplatelet activity by increasing intra-platelet cAMP. However, animal tests have also shown that *Glycyrrhiza glabra* L. activates pregnane X receptors (PXR) and increases the clearance of warfarin, thereby reducing its anticoagulant effect, so the mechanism of interaction between *Glycyrrhiza glabra* L. and warfarin requires further investigation.

#### Induction of Cytochrome P450 Enzyme Activity and Reduction of Warfarin Anticoagulation

*Hypericum perforatum* (Guanyejinsitao): The dried above-ground part of *Hypericum perforatum* L., family *Garciniaceae* has the effects of soothing the liver and relieving depression, clearing heat and dampness, reducing swelling and clearing milk, and can be used to relieve mild and moderate depression, and its main active ingredient is hypericin. Combined with warfarin it significantly induces the apparent clearance of S- and R-type warfarin and attenuates its anticoagulant effect (Jiang et al., 2004). *Hypericum perforatum* can induce the activity of CYP3A4 through the PXR (Wang et al., 2009), and induces the expression of CYP1A2 (Silva et al., 2016), promotes the metabolism of warfarin and weakens its anticoagulant effect.

*Panax ginseng* C.A.Mey. (Renshen) and *Panax quinquefolius* L. (Xiyangshen): The dried roots of *Panax ginseng* C.A.Mey. of the family *Pentacostaceae* have the effects of invigorating qi, promoting body fluid, soothing the nerves, and nourishing the mind. The modern pharmacological effects include anti-shock, increasing the amplitude of heartbeat and heart rate, anti-fatigue, promoting hematopoiesis, regulating cholesterol metabolism, enhancing the immune function of the body, and lowering blood sugar. Xiyangshen is the root of *Panax quinquefolius* L., a plant in the *Araliaceae* family, with the effects of tonifying qi and nourishing yin, clearing heat and generating fluid, improving immunity, inhibiting platelet coagulation, promoting blood circulation, and preventing arterial atherosclerosis. You et al. (2015) showed that these two herbs combined with warfarin decreased the INR in patients. *Panax ginseng* C.A.Mey. and *Panax quinquefolius* L. contain a variety of ginsenosides, of which ginsenoside F1 may induce the gene expression of CYP3A4 and enhance its enzymatic activity by activating the progesterone receptor in a concentration-dependent manner. However, it has also been shown (Malati et al., 2012) that ginsenosides Rg3, Rh2, and C-K inhibit the activities of CYP2C9, CYP3A4, and CYP2C19, while Rb1 and C-K moderately inhibit the activity of CYP1A2, and low doses of ginseng do not alter the activities of CYP3A4 and P-glycoprotein (Janetzky and Morreale, 1997; Hao et al., 2008). There are mixed findings in the literature regarding the effects of ginseng on CYP450 enzymes, and the mechanism by which it attenuates the anticoagulant effect of warfarin remains to be further clarified.

*Ginkgo biloba* L. (Yinxinye): *Ginkgo biloba* L. can induce hepatic cytochrome P450 enzymes, accelerate the metabolism of warfarin, thereby reducing the anticoagulant effect of warfarin (Zhang et al., 2007), and its mechanism of action is still unclear.

### DISCUSSION

**FIGURE 1:**
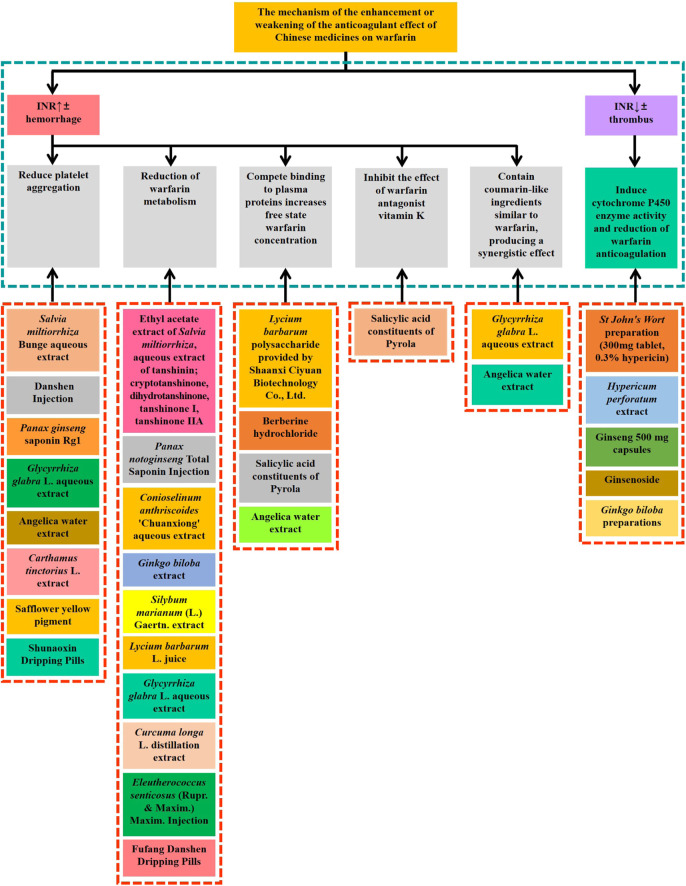
Chinese medicines (single Chinese medicine, Chinese medicine extract, Chinese patent medicine or compound medicine) that have influence on warfarin and their action mechanism.

Warfarin, is the oldest oral anticoagulant and is the most commonly used drug for patients requiring long-term anticoagulation therapy. The composition of Chinese medicine is complex, and the combination of Chinese medicine and warfarin during clinical treatment often leads to the occurrence of adverse reactions and can even endanger patients’ lives. Thus, clinicians and pharmacists need to have knowledge on the interactions between warfarin and Chinese medicine to avoid the occurrence of adverse reactions as much as possible and promote the rational use of clinical drugs. In this article, by reviewing a large number of literature studies, we have concluded that the five factors of clinically used Chinese herbal medicines affecting the mechanism of action of warfarin (Figure 1) are as follows:

Influences platelet aggregation response: *Salvia miltiorrhiza* Bunge increases the activity of coagulation factor III and fibrinolysis, and the tanshinone component of *Salvia miltiorrhiza* Bunge, *Carthamus tinctorius* L. extract, and Shunaoxin Dripping Pills can increase the peak concentration of warfarin and enhance its anticoagulant effect.

Affects warfarin metabolism: *Salvia miltiorrhiza* Bunge, *Silybum marianum* (L.), Gaertn. Flavonoids, *Conioselinum anthriscoides* ‘Chuanxiong’, *Glycyrrhiza glabra* L., and *Eleutherococcus senticosus* (Rupr. & Maxim.) Maxim. injection inhibit CYP450 enzyme activities, slowing warfarin metabolism. *Curcuma longa* L. promotes the synthesis of prostaglandin PGI2, reduces TXA2 production and interferes with intra-platelet cAMP or Ca^2+^ production; it has a strong inhibitory effect on the CYP3A isoform of the CYP45016 enzyme system, and may enhance the anticoagulant effect of warfarin.

Affects warfarin plasma protein binding rate: By replacing warfarin in the warfarin-albumin complex, *Salvia miltiorrhiza* Bunge, Tanshinone IIa, *Coptis chinensis* Franch. and *Phellodendron amurense* Rupr. contain berberine components, which significantly reduce the plasma protein binding rate of warfarin, increase the concentration of free warfarin in blood, and enhance the anticoagulant effect, of which tanshinone IIa and oleanolic acid have a certain inhibitory effect on coagulation factor Xa.

Affects the action of the warfarin antagonist vitamin K: *Pyrola calliantha* Andres calliantha Andres contains salicylates, which inhibit the oxidoreductase enzyme of vitamin K and hinder its metabolic cycle, reducing the absorption of vitamin K and affecting thrombin synthesis. *Tribulus terrestris* L. and its preparations can inhibit the production of vitamin K by intestinal flora and interfere with platelet aggregation, and affect the absorption, distribution, and metabolism of warfarin, resulting in an enhanced anticoagulant effect of warfarin, and the mechanism of action needs to be further investigated.

Contains coumarin-like components similar to warfarin: Angelica contains coumarin-like active ingredients such as parsley oil methyl ether, psoralen and bergamot lactone, which can inhibit thromboxane formation and platelet aggregation; it is also possible that warfarin can displace angelica from its binding to plasma proteins, which elevates the blood concentration of coumarin-like ingredients; ferulic acid in angelica can inhibit PAF and platelet aggregation reaction when administered *in vivo* or *in vitro*, which enhances warfarin’s anticoagulant effect. The coumarin derivatives in *Glycyrrhiza glabra* L. may show anti-platelet activity by increasing intra-platelet cAMP; *Glycyrrhiza glabra* L. activates pregnane X receptors and increases the clearance of warfarin, thus reducing its anticoagulant effect, so the mechanism of interaction between *Glycyrrhiza glabra* L. and warfarin requires further study.

Reducing the anticoagulant effect of warfarin is mainly through the induction of cytochrome P450 enzyme activity. *Hypericum perforatum* can diminish its anticoagulant effect by inducing CYP3A4 activity through PXR receptors and inducing CYP1A2 expression, which promotes warfarin metabolism. Ginsenoside F1 components contained in *Panax ginseng* C.A.Mey. and *Panax quinquefolius* L. induced CYP3A4 gene expression and enhanced its enzymatic activity, but ginsenosides Rg3, Rh2, and C-K inhibited CYP2C9, CYP3A4, and CYP2C19 activities, while Rb1 and C-K moderately inhibited CYP1A2 activity, and low-dose ginseng did not alter CYP3A4 and P-glycoprotein activity. The current literature is divided on the effect of ginseng on CYP450 enzymes, and the mechanism by which it attenuates the anticoagulant effect of warfarin remains to be further clarified.

The mechanism of the interaction between warfarin and herbal medicines has been basically clarified by cytological, pharmacodynamic and pharmacokinetic studies, and the following problems were found during the collation of this review: Some herbal medicines have inconsistent conclusions between *in vitro* and *in vivo* experiments, pharmacology and clinical study results. For example, clinical studies showed that Danshen drops and warfarin have synergistic effects on anticoagulation, but some animal experiments showed no significant changes in the pharmacodynamics and pharmacokinetics of warfarin when compound Fufang Danshen Dripping Pills were taken with warfarin; coumarin derivatives in *Glycyrrhiza glabra* L. may show antiplatelet activity by increasing intra-platelet cAMP, but some animal experiments showed that *Glycyrrhiza glabra* L. can activate pregnane X receptors and increase the clearance of warfarin, thus decreasing its anticoagulant effect. However, some animal tests have shown that *Glycyrrhiza glabra* L. activates progesterone receptors and increases the clearance of warfarin, thereby decreasing its anticoagulant effect; the literature also shows inconsistent conclusions regarding the effects of *Panax ginseng* C.A.Mey. and *Panax quinquefolius* L. and GBE on CYP450 enzymes. The data on the interaction between warfarin and Chinese herbal medicines are not comprehensive, for example, clinical studies have been conducted on *Lycium barbarum* L., *Hypericum perforatum* and *Tribulus terrestris* L., but no pharmacological studies have been conducted. Pharmacological studies have been conducted on *Eleutherococcus senticosus* (Rupr. & Maxim.) Maxim. injection, but no clinical studies have been performed and no reports are available. Therefore, in the future, we should focus on the interaction between this type of clinically-used Chinese medicine and warfarin in order to obtain better research theories and data to guide the rational and safe use of clinical drugs.

### CONCLUSION

With the improvement in living standards, elderly patients who have been taking warfarin for a long time and who pay attention to TCM health care often take TCM on their own, and warfarin has the characteristics of a long half-life and a narrow therapeutic window, and drug interactions are the main cause of warfarin adverse events. The composition of TCM is complex, and the same TCM may have dual effects of enhancing and attenuating the anticoagulant effect of warfarin, and the INR values of patients should be monitored while they are receiving warfarin. For the herbal medicines mentioned in this paper that interact with warfarin, TCM practitioners or TCM pharmacists should screen patients when using warfarin and adjust their herbal medicine selection accordingly, strengthen the monitoring of the use of high-risk drugs, especially herbal medicines that activate blood circulation and resolve blood stasis**,** and adjust the dose of the drug according to the monitoring results to prevent or reduce the occurrence of adverse reactions and improve the effectiveness and safety.

## Abbreviations

ADP, adenosine diphosphate; AUC, area under drug time curve; cAMP, cyclic adenosine monophosphate; CYPs, cytochrome P450s; 5-HT, 5-hydroxytryptamine; GBE, *Ginkgo biloba* extract; INR, international normalized ratio; LBP, *Lycium barbarum* polysaccharides; PAF, platelet activating factor; PGI2, prostaglandin 2; PNS, *Panax notoginseng* saponins; PT, prothrombin time; PXR, pregnane X receptor; SAA, salvianolic acid A; 6-keto-PGFla, 6-keto-prostaglandin; TCM, Traditional Chinese Medicine; TT, thrombin time; TXA-2, thromboxane A-2; TXB2, thromboxane B2.
